# Controlled human malaria infection studies: insights into recent advances and key immunological and ethical implementation lessons

**DOI:** 10.3389/fimmu.2025.1672945

**Published:** 2025-09-18

**Authors:** Rodney Ogwang, Mohamed Adan, Philip Bejon, Melissa C. Kapulu

**Affiliations:** ^1^ Biosciences Department, Centre for Geographic Medicine Research (Coast), Kenya Medical Research Institute-Welcome Trust Research Programme, Kilifi, Kenya; ^2^ College of Health Sciences, Makerere University, Kampala, Uganda; ^3^ Department of Modernising Medicine, Nuffield Department of Medicine, University of Oxford, Oxford, United Kingdom; ^4^ Centre for Tropical Medicine and Global Health, Nuffield Department of Medicine, University of Oxford, Oxford, United Kingdom

**Keywords:** human infection studies, *Plasmodium falciparum*, vaccines, monoclonal antibodies, immunity, correlate of protection, Africa

## Abstract

Controlled human infection studies offer a unique opportunity to study the efficacy of novel interventions, mechanisms of infection and disease, as well as determine correlates of protection that may underpin the development of novel interventions. Controlled human malaria infection (CHMI) studies supported the clinical development of the first malaria vaccines (i.e. RTSS/AS01 and R21/Matrix-M). The CHMI model accurately predicted efficacy of these vaccines and accelerated their clinical development. In addition to vaccine development, over the last decade CHMI studies have supported the advancement of drugs, monoclonal antibodies (mAbs) and been instrumental in characterising immunity to malaria by unravelling immunological and innate mechanisms that may mediate protection. Here, we briefly review the history and rationale of the available falciparum malaria CHMI models. We highlight key applications and lessons learned from CHMI studies conducted in naïve and endemic populations with respect to immunological advances, discoveries in therapeutic targets such as mAbs, and transferring of the models from high income to low- and middle-income settings.

## Introduction

Malaria remains a global health concern as half the world’s population remains at risk of infection (1, 2). The World Health Organization (WHO) estimates ~263 million malaria cases with ~597–000 deaths occurred in 2023 (3) rising by ~11 million cases compared to 2022. The increasing reports of malaria resurgence in Africa (4), despite implementation of the available control tools, makes the historic approval of the first malaria vaccines (RTSS/AS01 in 2021 and R21/Matrix-M in 2023) highly welcomed (5, 6). However, the level of vaccine protection offered by these vaccines is incomplete (7). Therefore, continued investment in research and development is important including other pre-erythrocytic vaccines (e.g. whole sporozoite vaccines), efforts to develop and implement either multistage vaccines (e.g. including erythrocytic or transmission blocking components) or therapeutic interventions such as monoclonal antibodies (mAbs).

Malaria elimination will likely require multiple interventions, including currently established approaches (vector control strategies – such as indoor residual spraying with effective insecticides, and the use of long-lasting insecticide treated bed nets, plus the accurate diagnosis and treatment with appropriate antimalarial drugs (8, 9)) and additional tools such as the vaccines R21/Matrix -M and RTS,S/AS01, that are currently being deployed in a number of African countries, specifically in areas of high malaria transmission (7). Emerging interventions such as fast acting mAbs and long-acting drugs may also contribute to future malaria control (10). Controlled human infection studies provide considerable utility for the clinical development and licensing of new interventions (11, 12). Challenge studies may be used to derisk costly phase III trials (13). For example, the clinical development and licensure of Vaxchora (for cholera) (14) and Vi-tetanus toxoid conjugate vaccine (for *Salmonella typhi*) (15) benefited from controlled human infection studies. In addition, challenge studies provide a platform to study disease pathogenesis, aetiology, natural history, acquired immunity, and immune correlates/surrogates of protection that may further guide intervention design (11, 13).

Human infection studies involve the experimental exposure of an individual (mainly healthy adults) to a disease- and/or infection-causing microbe (parasite, virus or bacteria) in a highly controlled setting for scientific merit (13). Since their practice in the Middle East during the early modern era (and likely earlier) and further uptake by Edward Jenner (while studying smallpox) in the early 18^th^ century, important scientific lessons have been gained across the >30 disease models developed to date (16). For malaria, the controlled human malaria infection studies (CHMI) have played key roles in vaccine and drug development such as the advance of the now approved RTS, S/AS01 and R21/Matrix M malaria vaccines (17, 18) and drugs such as atovaquone–proguanil (19) and Artefenomel (20). CHMI studies have also been a source of discovery and development for mAbs (21, 22), genetically attenuated parasites (GAPs) (23–25) and chemoprophylaxis vaccination with sporozoites (CVac) using chloroquine (late arresting parasites for improved liver stage T cell immunity) (26–28).

**Figure 1:**
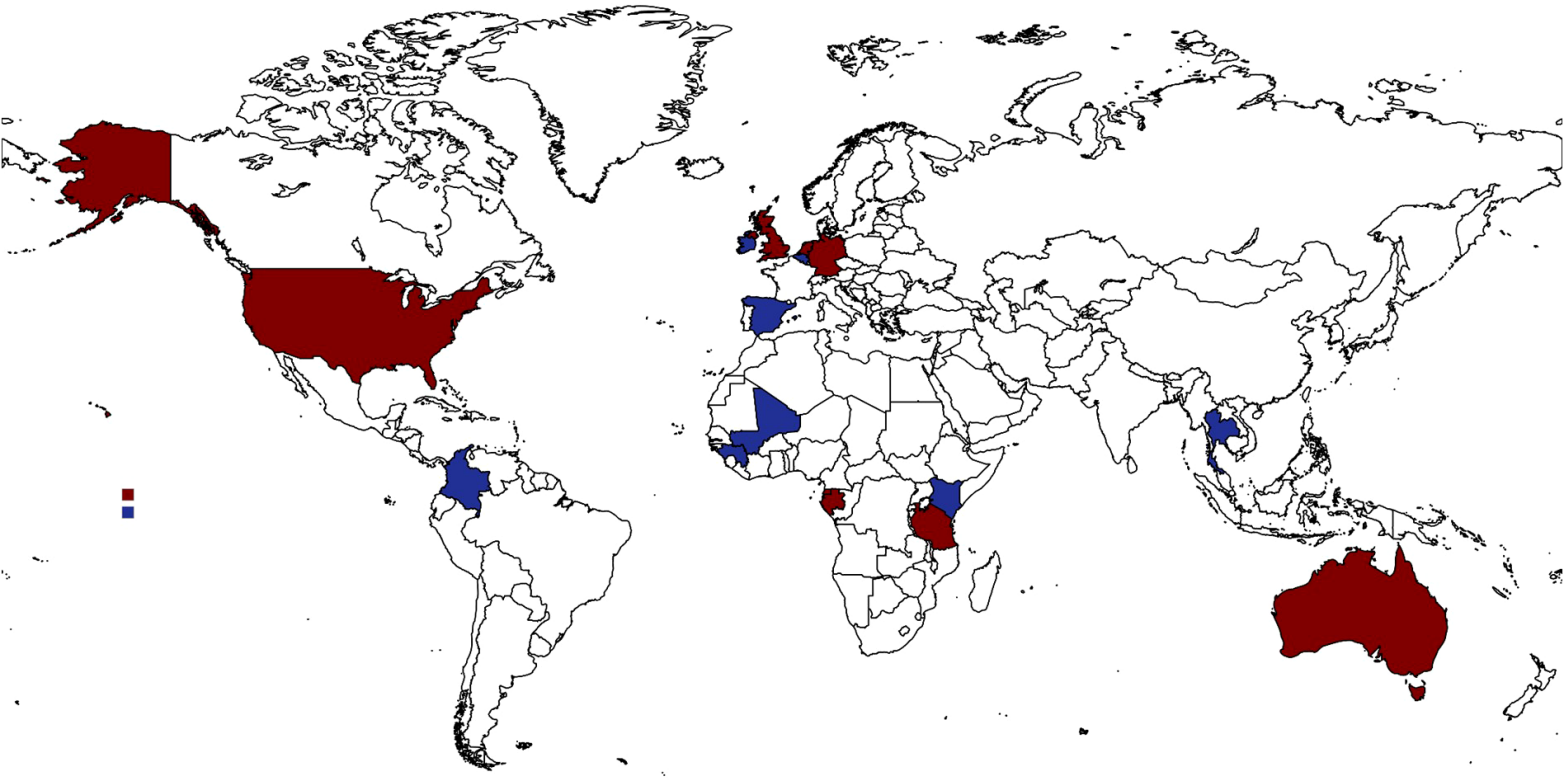
Global status of CHMI studies. Map of countries where CHMI studies have been conducted in both the global north and global south. Red represents where more than five CHMI study protocols have been undertaken, and blue represents countries where five or less CHMI protocols have been undertaken. This represents studies of all malaria parasite strains including falciparum (Australia, Belgium, Equatorial Guinea, Gabon, Gambia, Germany, Kenya, Mali, Spain, Tanzania, The Netherlands, UK, USA); malariae (Australia) and vivax (Australia, Colombia, Thailand, and USA).

Despite the burden of malaria being greatest in Africa, the majority of CHMI studies have been conducted in malaria naïve volunteers in high-income countries (29). The reasons for this include: (a) lack of availability of challenge agents suitable for delivery in endemic settings; (b) hesitance to conduct research on apparently “vulnerable” populations in LMICs; (c) lower research infrastructure and capacity; (d) funding availability; and (e) ethical and regulatory challenges. Despite these, the first modern CHMI (well-designed and ethical) conducted in Africa occurred in 2012 in Tanzania followed by a study in Kenya the following year using the then newly developed, aseptic, purified, cryopreserved *Plasmodium falciparum* sporozoite product, Sanaria^®^ PfSPZ Challenge (NF54) (30, 31). Since then, multiple studies have occurred, and several lessons and insights have been gained thus changing the global landscape and capacity for the conduct of CHMI studies (**Figure 1**).

Here, we briefly review the history and rationale of the different CHMI models. We then summarise the immunological and ethical lessons learned from CHMI studies conducted among semi-immune individuals in Africa.

## Design of controlled human malaria infection models

The discovery of mosquitoes as the vector of the *Plasmodium* parasite by Grassi, Bignami and Ross may be considered the beginning of CHMI studies (32–34). They fed mosquitoes on hospitalized malaria patients, then after allowing the mosquitoes sufficient time to become infectious, they used these mosquitoes to infect healthy volunteers, thus confirming mosquitoes as the malaria transmission vector (35). Deliberate human infections with malaria then grew in prominence with the development of malariotherapy (the use of malaria-induced fever for the treatment of neurosyphilis) which earned Julius Wagner-Jauregg the Nobel prize in 1927 (36–38). Malariotherapy strengthened our understanding and later acceptance of controlled human malaria infection until its use waned after the discovery of antibiotics in the 1940s (36, 37).

The first modern-day controlled human malaria infection (CHMI) experiment in healthy volunteers was conducted by researchers of the Walter Reed Army Institute and the Naval Medical Research Institute in the USA in 1986. Since then, >80 CHMI studies have been conducted. Modern CHMI involves the intentional infection of healthy volunteers (adults) with malaria parasites prepared under highly regulated Good Manufacturing Practice (GMP) guidelines. In the modern era, volunteers only participate after appropriate screening and full informed consent. Individuals are followed daily to a pre-determined density of blood-stage parasites, or a clinical symptomology define endpoint where curative doses of appropriate anti-malarial drugs are provided (39, 40). The monitoring of blood parasitemia was initially carried out by thick blood smear microscopy, however in recent times the more sensitive 18s ribosomal quantitative polymerase chain reaction (qPCR) assays are preferred (41–43). Among CHMI studies conducted to date no severe malaria symptoms have been reported, although acute myocarditis with full recovery probably linked to PFSPZ infection by mosquito bite under chloroquine chemoprophylaxis has been described in two volunteers (44, 45). Importantly, no deaths or lasting disabilities have ever occurred as a result of CHMI.

## Controlled human malaria infection reagents, models and applications

**Figure 2:**
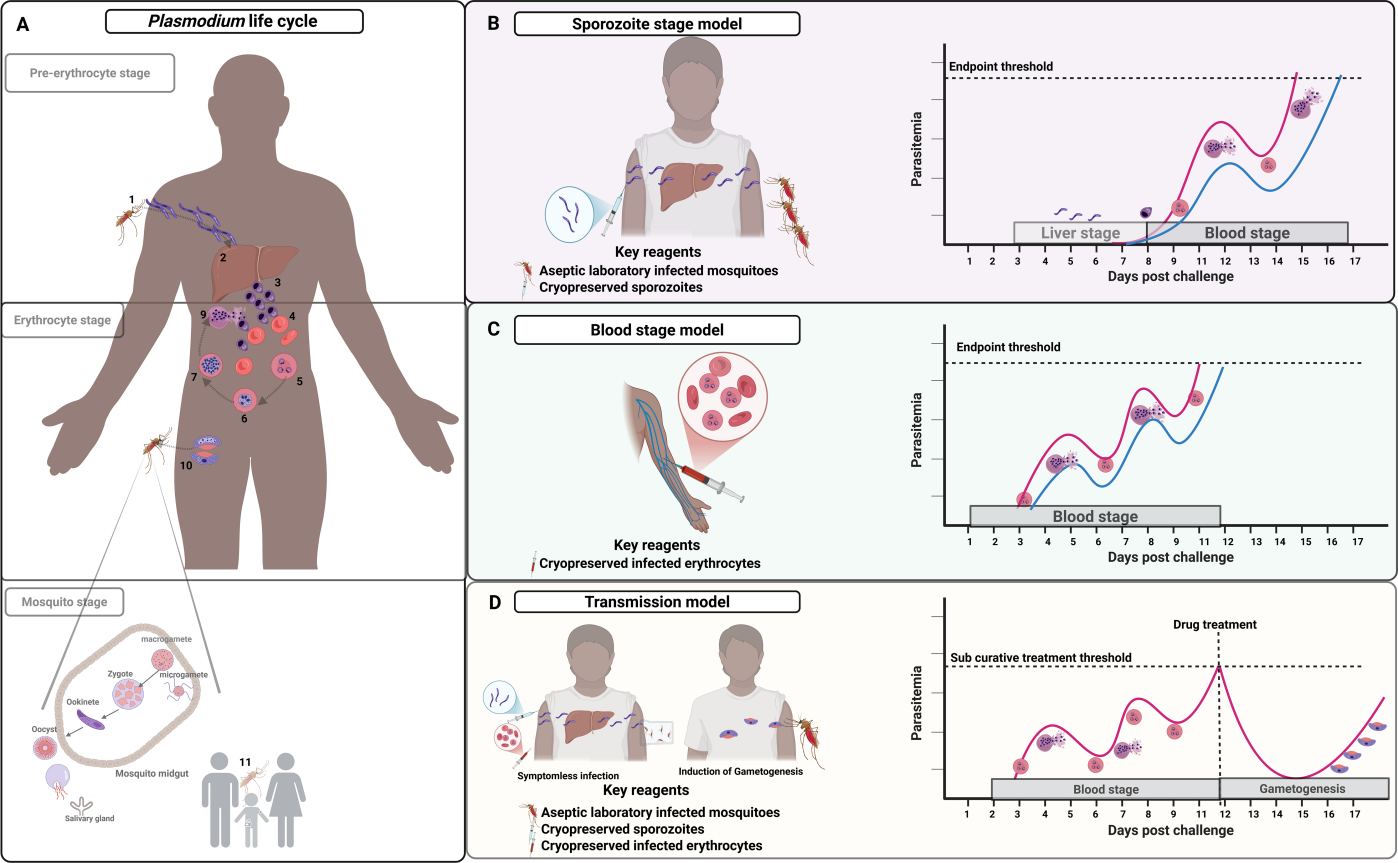
*Plasmodium falciparum* challenge models in relation to life cycle stages. CHMI models are currently set up to cover and replicate the pre-erythrocytic, erythrocytic, and mosquito stages life cycle stages. **(A)** shows the natural life cycle involving: mosquito injection of sporozoites (1); liver hepatocyte development into liver stage schizonts which release merozoites into blood circulation (2); merozoites invade red blood cells initiating the erythrocytic stage (3-4); continuous ring stage to trophozoite differentiation and schizont stage which ends with red cell rupture releasing newly formed erythrocytic merozoites (5-9); leading to sexual gametocytes and uptake by mosquitoes (10); and male and female gametocyte fertilization and onward transmission of sporozoites (11). **(B)** shows the sporozoite model with injection of sporozoites (either aseptic laboratory infected mosquitoes or cryopreserved sporozoites) then parasite monitoring from day 6 post-infection (red line for naïve individuals and blue line semi-immune). **(C)** shows a blood-stage model with injection of infected erythrocytes and parasite monitoring typically from day 2 post-infection; and **(D)** shows the transmission model involving sub-curative drug treatment following infection (either sporozoite or blood-stage) at pre-defined parasitaemia threshold to promote gametocyte development with transmission experiments either *ex vivo* or direct skin mosquito feeding. In all the models, the endpoint is pre-specified: symptoms and/or set parasitaemia threshold (e.g. 500 parasites/μl).

In summary, the malaria life cycle begins with anopheline mosquitoes injecting sporozoites into the skin or directly into blood vessels during blood feeding. Sporozoites then invade liver cells and develop into merozoites that are released into blood. Merozoites invade blood cells to form trophozoites, and a continuous cycle of asexual replication leads to successive generations of merozoites that re-invade red blood cells, potentially leading to exponential parasite growth. It is at this point that disease symptoms begin to present. A subset of merozoites differentiate into sexual forms (gametocytes) that infect mosquitoes for onward transmission (**Figure 2**).

To achieve safe, ethical and reproducible infection of volunteers, initially high quality and then primary GMP level reagents were developed. These included non-aseptic and aseptic laboratory-reared *Plasmodium* infected mosquitoes; and aseptic, purified, cryopreserved *P. falciparum* sporozoites (PfSPZ Challenge), that underpin the sporozoite model. Aseptic *Plasmodium* infected erythrocytes have been developed to support the blood stage models. (**Table 1**).

**Table 1:** CHMI parasite species and strain reagents.

Parasite strain/species	Model reagent available	Mosquito species	ref
*P. falciparum*, NF54/3D7 (West Africa)	Mosquito, sporozoite, iRBC	*An. stephensi, An freeborni*	(46, 47)
*P. falciparum*, NF135.C10 (Cambodia)	Mosquito and sporozoite	*An. stephensi*	(48)
*P. falciparum*, NF166.C8 (Guinea)	Mosquito and sporozoite	*An. stephensi*	(49)
*P. falciparum*, 7G8 (Brazil IMTM22 isolate)	Mosquito and sporozoite	*An. stephensi*	(46)
*P. falciparum*, Ethiopian (Tamenie) strain	Mosquito		(50, 51)
*Plasmodium knowlesi*	Sporozoite		(52)
*P. vivax*, natural isolate (Colombia)	Mosquito	*An. albimanus*	(53, 54)
*P. vivax*, natural Isolate (Thailand)	Mosquito	*An. dirus*	(55, 56)
*P. malariae isolate*	iRBCs	N/A	(57)
*Plasmodium falciparum* (Uganda I)	iRBCs	N/A	(58)
*P. falciparum HMP02* (an isolate from Ghana)	iRBCs	N/A	(59)
*P. vivax* cell bank (Australia)	iRBCs	N/A	(59, 60)
*P. vivax Thai PvW1 clone*	iRBCs	N/A	(61, 62)

iRBCs, infected red blood cells; N/A, not applicable.

## Controlled human infection reagents

Malaria challenge experiments can be conducted with a range of challenge agents:

1. Laboratory-reared *Plasmodium* infected mosquitoes: Early studies relied on mosquitoes fed on malaria infected volunteers or their collected blood samples. This approach had safety, reproducibility, and efficiency concerns (63). Further, the availability of gametocytemic *P. falciparum*-infected people with which to infect mosquitoes is a limiting resource. Consequently, to facilitate safer volunteer infections, methods for rearing, aseptic *P. falciparum* sporozoite-infected mosquitoes under GMP were developed (64, 65). This involved the methods for the determination of sporozoite loads, confirmation of malaria transmission, and the avoidance of mosquito co-infections (64, 65). An important development was the growth of continuous *in-vitro* culture methods of *P. falciparum* (66, 67), and blood feeding of laboratory grown mosquitoes (68). These eliminated the need for blood from infected patients and increased access to infected mosquitoes. This approach became the mainstay approach for malaria infection and multiple parasite strains were developed and utilized for human infection using these methods (**Table 1**). All such studies have been done with non-aseptic mosquitoes except for two trials with aseptic *A. stephensi* mosquitoes (39, 64).
2. Aseptic, purified, cryopreserved sporozoites: Scientists at Sanaria Inc in Maryland USA advanced methods to produce aseptic, purified, cryopreserved viable PfSPZ (69). This effort was motivated by developing whole sporozoite based vaccines, but in addition has significantly increased the potential for CHMI studies as aseptic, purified, cryopreserved sporozoites could be stored and transported across sites with relative ease, and can be injected with needle and syringe without the requirement for mosquito containment facilities. This has particularly been important for facilitating the first CHMI studies in Africa, beginning in Tanzania (70), and Kenya (71) only two years after the first CHMI study with PfSPZ Challenge (NF54) in the Netherlands (31). To date CHMI studies have been conducted in Mali (72), Gabon (73), Gambia (74) and Equatorial Guinea (75).
3. Aseptic *Plasmodium* infected erythrocytes: Early accounts of direct blood infection studies have been reported and suffer ethical and safety concerns (76, 77). However, in the modern era GMP grade aseptic *Plasmodium* infected erythrocytes stocks were generated by researchers in Australia at the Queensland Institute of Medical Research (QIMR) (78). Two donors were deliberately infected with *P. falciparum* 3D7 via mosquito bite and blood aliquots of cryopreserved erythrocytes prepared. Blood samples were extensively tested to ensure the safety to potential volunteers (78). Direct infection using blood stage parasites hinges on the availability of blood stocks collected from suitable malaria-infected “universal” bloodxgroup O Rh D-negative blood doners (79) which may be a finite resource. The alternative approach is to generate stocks from laboratory culturing of large volumes of defined *P. falciparum* isolates in blood group O Rh D-negative blood at GMP levels facilities. This is followed by characterization cryopreservation in appropriate aliquots (46).

## The mosquito bite model

Aseptic laboratory reared mosquitoes infected by membrane feeding approaches using gametocyte-stage parasites from laboratory culture (standard membrane feeding assays (SMFAs)), are used to challenge consenting volunteers (68). Typically, three-five infected mosquitoes are allowed to feed on volunteers for 5 mins (63, 80, 81). Parasitaemia is then monitored either by microscopy or qPCR to study endpoints as described above (**Figure 2B**). For *P. vivax* challenge studies however, continuous *in vitro* culture is not yet established, therefore the *P. vivax* model is limited to the use of clinically infected individuals for direct mosquito infection or to provide blood aliquots that can be used to feed mosquitoes, as was previously the case for falciparum. On the other hand, gametocytemia is more frequent and earlier to develop in *P. vivax* infection, and so this requirement is somewhat less limiting than was historically the case for falciparum (82). The mosquito challenge model has been the hallmark of CHMI studies and supported the majority of CHMI studies (**Table 2**). Using this approach >2,000 human volunteers have been challenged safely.

**Table 2:** Efficacy in CHMI of vaccine and monoclonal antibody candidates tested to date.

Vaccine/mAb candidate/Antigen		Challenge approach/agent	Efficacy	Setting	Ref
Whole vaccines	PfSPZ Vaccine	Mosquito bite	100%	HIC	(81, 83–86)
		PfSPZ Challenge	50-100%^a^	LMIC	(70, 72, 73, 75)
	PfSPZ-CVac	Mosquito bite	100%	HIC	(28)
		PfSPZ Challenge	100%	LMIC	(27)
	PfSPZ-GA1	Mosquito bite	12%	HIC	(23)
	PfSPZ-GA2	Mosquito bite	89%	HIC	(25)
	PfGAP3KO vaccine	Mosquito bite	50% & 15% ^b^	HIC	(87)
	Whole infected erythrocytes with therapy	*P. falciparum* iRBCs	75%	HIC	(88)
Single recombinant Antigens	RTSS/AS01	Mosquito bite	50-86.7% ^a^	HIC	(17, 89, 90)
	RTS, S/AS02A	Mosquito bite	33-45% ^a^	HIC	(90–92)
	RTS, S QS21	Mosquito bite	0%	HIC	(93)
	RTS, S and ME-TRAP with AS02	Mosquito bite	0%	HIC	(94)
	ICC-1132 with Seppic ISA 720	Mosquito bite	0%	HIC	(95)
	R21/Matrix M	Mosquito bite	82%	HIC	(18)
		PfSPZ Challenge	0-100%^c^	LMIC	(96)
	CelTOS (FMP012/GLA-SE)	Mosquito bite	0%	HIC	(97)
	ME-TRAP with AS02	Mosquito bite	0%	HIC	(94)
	DNA/MVA ME-TRAP	Mosquito bite	0%	HIC	(98)
	ChAd63-MVA CS	Mosquito bite	7%	HIC	(99)
	ChAd63-MVA ME-TRAP	Mosquito bite	17%	HIC	(99)
	ChAd63-MVA ME-TRAP	PfSPZ Challenge	0%	LMIC	(96)
	LSA-1 with AS01 or AS02	Mosquito bite	0%	HIC	(100)
	PfCS102 Montanide ISA 720	Mosquito bite	0%	HIC	(101)
	ChAd63-MVA AMA-1	Mosquito bite	0%	HIC	(102)
	ChAd63-MVA MSP1	Mosquito bite	0%	HIC	(102)
	RH5.1/AS01B	*P. falciparum* iRBCs	0% ^d^	HIC	(103)
	AMA-1/AS01B and AMA-1/AS02A	*P. falciparum* iRBCs	0%	HIC	(104)
	AMA-1 Vaccine (FMP21/AS01)	*P. falciparum* iRBCs	0%	HIC	(105)
	AMA1-C1/Alhydrogel + CPG 7909	*P. falciparum* iRBCs	0%	HIC	(106)
Combination vaccines	FFM ME-TRAP+PEV3A	Mosquito bite	0%	HIC	(107)
	FFM ME-TRAP	Mosquito bite	0%	HIC	(108)
	DNA/Ad CSP and AMA1 (NMRC-M3V-D/Ad-PfCA Vaccine)	Mosquito bite	27%	HIC	(109)
	Ad-NMRC-M3V-Ad-PfCA vaccine (CSP and AMA1)	Mosquito bite	0%	HIC	(110)
	RTS,S/AS01B with ChAd63-MVA ME-TRAP	Mosquito bite	75 - 82.4% ^a^	HIC	(111)
	ChAd63-MVA MSP1 and AMA1	Mosquito bite	0%^d^	HIC	(102)
	MSP2/MSP1/RESA in Montanide ISA 720 adjuvant (SEPPIC).	*P. falciparum* iRBCs	0%	HIC	(112)
	GMZ2 (GLURP and MSP3)/CAF01 or Alhydrogel adjuvanted	PfSPZ Challenge	<25%	LMIC	(113)
mAbs	Monoclonal antibody CIS43LS	Mosquito bite	82%	HIC	(114)
	Monoclonal antibody L9LS	Mosquito bite	88%	HIC	(115)
	Monoclonal antibody MAM01	Mosquito bite	TBD^e^	HIC	(116)

PfSPZ Challenge (aseptic, purified, cryopreserved sporozoites); PfSPZ C-Vac (*Plasmodium falciparum* (Pf) Sporozoite (SPZ) Chemoprophylaxis Vaccine); ^a^Range observed within different studies. ^b^50% at primary challenge and 16% when secondary challenge 6 months later. ^c^0% was observed with DVI and 100% with intradermal administration of PfSPZ Challenge, ^d^delayed time to parasitemia ^d^Sterilisaing protection observed in 1 volunteer,^e^TBD – to be done.

## Cryopreserved sporozoites

In this model, aseptic purified and cryopreserved sporozoites can be thawed and injected into human volunteers via intravenous, intramuscular or subcutaneous injections at different doses of choice (117, 118) (**Figure 2B**). The mosquito bite challenge closely recapitulates the natural course of infection compared to the PfSPZ infection model. In general, it has taken exposure to the bites of five non-aseptic, infected mosquitoes inoculating an estimated 15 to 500 Pf sporozoites to achieve 100% infection of non-immune recipients (119). In contrast it takes administration of 3,200 aseptic, purified PfSPZ via direct venous inoculation to achieve 100% infection of non-immune recipients (118). This is due to the loss of infectivity associated with cryopreservation of PfSPZ. However, it has provided considerable utility in the study of vaccine candidates, drugs and biologics and acquired immunity.

CHMI of PfSPZ with needle and syringe provides an exact quantifiable dose of inoculum and allows good control, quality assurance of CHMI that do not depend on infection, rearing and biting of infected mosquitoes that are needed for direct mosquito infections. Furthermore, mosquito bite challenge currently depends on the vector *Anopheles stephensii*, which has become an invasive vector in some parts of Africa and so would be problematic to import and implement (120).

## Blood stage model

The blood-stage challenge model involves the intravenous administration of aseptic *Plasmodium* infected erythrocytes, leading to a blood stage infection in the absence of pre-erythrocytic stages (78, 79, 121). This is typically a few hundred to 2,500, parasites. Blood-stage parasite multiplication is then monitored by microscopy or qPCR (41). This continues until a defined parasite threshold or patient symptomology when curative doses of antimalarial drugs are administered (**Figure 2C**). This model skips the liver stages of disease and would not allow study of pre-erythrocytic immunity such as the effect of circumsporozoite antibodies. It does however offer control over the starting blood stage parasite burden therefore enabling comparisons and study of parasite growth rate, whereas the sporozoite based challenge models result in greater variability in the number of merozoites exiting the liver stage, which may complicate precision in studying growth rates (122). Another advantage of the blood-stage challenge model is that it allows longer follow-up periods, due to low inoculation dose compared to the number of merozoites released from an infected hepatocyte(s).

## Transmission-blocking model

This model was developed to study the transmission of gametocytes from an infected individual to mosquitoes (123, 124). The premise is the need to understand transmission blocking interventions such as vaccines, drugs and monoclonal antibodies (mAbs) that may be important for malaria elimination particularly to enable control of residual transmission on the path to elimination (125).

First, symptomless malaria infection of a volunteer is induced. This can be achieved by using any of the infection models (i.e mosquito bites, cryopreserved sporozoites or infected blood cells), and providing antimalarial drugs in non-curative doses to suppress parasitemia while allowing the development of sexual stage parasites (**Figure 2D**). In *P. falciparum* infections, sexual parasites appear later in the time course of infections. Sub curative doses of antimalarials drugs (such as sulfadoxine-pyremethamine and or piperaquine), may stimulate the appearance of gametocytes in the peripheral circulation (123, 126). Finally, transmission is determined by feeding mosquitoes through membrane feeding devise or directly on skin. Fed mosquitoes are dissected and oocysts quantified to measure transmission potential (11, 127).

## Applications for controlled human malaria infection studies

Controlled human infections have been utilized for antimalarial drug assessment, vaccine and mAb efficacy estimation, and study of naturally acquired immunity and innate resistance to malaria (11). Other uses of the model include evaluating diagnostic tools and biomarkers, studying immune correlates and disease pathogenesis.

Antimalarial drug assessment: Drug resistance to commonly used antimalarial drugs is on the rise and is a major concern (3, 128). The development of new, long-acting and single dose treatments is therefore a research and public health priority. Following pre-clinical development and safety studies, CHMIs offers the opportunity to quickly evaluate the therapeutic activity, pharmacokinetic and pharmacodynamics properties of new drugs (19, 20). The highly controlled design of these studies allows appropriate drug efficacy reporting in non-immune participants without the potential overestimations of efficacy that may be seen in semi-immune adults, or the risks of first testing efficacy in children. Both mosquito bite- and blood-stage-initiated infections have been used to study antimalarial drugs (46). However, the blood stage approach may be favored as it allows control of the initial parasite burden.
Vaccine and monoclonal antibody efficacy estimation: RTS, S for example underwent a lengthy developmental phase spanning over 30 years, supported by efficacy data from CHMI studies (10). CHMI models allowed the optimization of vaccine dose, route, schedule, and adjuvant formulation prior to (or in some cases alongside) field trials. Similarly, R21/Matrix-M demonstrated early efficacy within CHMI studies that supported its rapid clinical development. Other recombinant vaccines candidates, DNA/viral vector-based vaccines and whole sporozoite vaccines have been evaluated in CHMI models providing important proof-of-concept efficacy data for go/no go development decision making (**Table 2**). Initial concerns were that CHMI studies may be too stringent for vaccine candidate evaluations and would not correlate with actual field efficacies. A reduction in efficacy within the field that probably relates to immunogenicity reductions in malaria-exposed children, waning of antibody levels, and heterogeneity in circulating parasites stains has been observed. While efficacy studies using CHMI models in naïve adults conducted in the US and UK for subunit vaccines have often translated to field efficacy it has not necessarily been the case for whole parasite approaches such as whole PfSPZ vaccine studies (129). It is therefore important to test vaccine candidates using CHMI models within malaria endemic populations. Hence two key considerations that may improve the accuracy of CHMI models in predicting efficacy are: a) conducting CHMI in endemic areas, therefore capturing the impact of potential reductions in immunogenicity with some vaccinations; b) developing new parasite strains to use as challenge agents in CHMI to capture the potential impact of diverse genotypes in the field.

More recently, monoclonal antibodies (mAbs) are undergoing clinical development as potential tools for malaria control. The highly potent and protective CIS43LS (21) and L9LS (22) mAbs were discovered in samples from individuals vaccinated by PfSPZ vaccine who were protected in sporozoite challenge studies. The efficacies of these mAbs were later tested within CHMI models and were shown to be highly protective, and efficacy for one mAb was recapitulated within clinical field trials in malaria endemic areas. Currently, field efficacy trials are ongoing to evaluate their utility in light of cost, production and implementation constraints (115, 130).

Whole sporozoites for vaccination: Here the immune system can be exposed to the entire array of parasite antigens as opposed to a single recombinant protein. Radiation attenuated, chemically and genetically attenuated parasites that arrest parasite growth are in different stages of clinical development (131). Radiation attenuated *PfSPZ* are most advanced and have demonstrated sterile protection (132, 133). Administration of PfSPZ under chemoprophylaxis (PfSPZ-CVac) involves infection through mosquito-bite or direct venous sporozoite induced infections under chemoprophylaxis. This has been conducted using anti-malarial drugs such as chloroquine, mefloquine and pyrimethamine, that prevent blood stage infection while allowing the completion of liver stage parasite development. Sterile protection against heterologous CHMI for 3 months has been observed with this approach. Genetically attenuated parasites that arrest development are also in development (PfSPZ-GA1 (23); PfSPZ-GA2 (25); PfGAP3KO vaccine (87)) with varying levels of efficacy. These parasite lines may be utilized as tools to study immunity (134, 135).
Whole blood stage vaccines have also been evaluated. Here parasitized red blood cells and are administered with each infection controlled with Malarone (atovaquone-proguanil) prior to patency (88). Protection was observed in 3 out of 4 volunteers, however residual anti-malarial drugs that were not seen in the control arm because of their lack of prior exposure may have been a significant confounder of the protection data (136).
Diagnostic development and evaluation: Finally, the fast, accurate, and cost-effective diagnosis of malaria using dipstick rapid diagnostic tools that target the Histidine rich protein-2 (HRP2) transformed diagnosis and treatment. However, the concerning emergence of HRP2 deleted parasites that render RDTs ineffective is worrying (137, 138). Although the parasite lactate dehydrogenase (pLDH) based test kits are available, the development of more sensitive, cheap, non-invasive tools is important (139, 140). CHMI provides a platform to test different diagnostic tools providing specimens with controlled timing of infection and monitoring. Examples of the utilization of CHMI for diagnostic development include, the evaluation of breath specimens for *Plasmodium falciparum* biomarkers and the development of *Plasmodium falciparum* HRP-2 antigen a rapid dipstick antigen-capture assay (141).
Study of acquired immunity: Early studies to understand immunity utilized *post-hoc* analysis of patients receiving malariotherapy and highlighted its slow acquisition over repeated exposures and its strain-dependent nature (36–38, 142, 143). More recently homologous repeat challenge experiments among naïve adults have been conducted to evaluate acquired immunity (144, 145). One study used a mosquito bite challenge model to induce four consecutive repeat malaria episodes with a homologous strain (NF54), where a delay in patent parasitemia was observed with each iterative infection (144). A CHMI study among Kenyan adults with different levels of malaria exposure showed differing clinical phenotypes and parasite growth patterns. A proportion of individuals from areas with higher exposure presented with no parasites or with slower parasite growth, while those from areas of less exposure presented with exponential parasite growth with early development of clinical symptoms (71). These together confirm the slow acquisition of immunity over repeated exposures. Another study utilized a blood stage model to induce three consecutive infections; however no significant anti-parasitic blood-stage immunity was observed in the majority of re-challenges (145). Though careful interpretation is required this may suggest earlier acquisition or a lower protection threshold for liver stage responses compared to blood stage. The key immunological mechanisms of antimalarial immunity are reviewed in detail elsewhere (146–149).

## Key lessons learned from CHMIs conducted in endemic populations

The earliest reports of deliberate human malaria infection in Africa occurred between 1954-1963. The first reported study was conducted in 1954 among Ugandan adults (58). This study showed the protective effect of sickle cell trait (SCT) against malaria. In the study, 30 volunteers (n=15 with SCT and n=15 controls) were infected by either parasitized red blood cells or exposure to infected mosquito bites. The individuals with SCT had lower parasite infection rates (2/15) and parasitemia compared with 14/15 in the control group (58). The same question was later addressed in modern CHMI studies using aseptic purified cryopreserved sporozoites administered by direct venous infection. This showed more nuanced findings with slower growth rates in individuals with SCT rather than complete protection as suggested in the 1954 study (73). In addition, modern CHMIs confirmed the protective effect of other blood genotypes in particular the Dantu blood group which had been seen to be protective in GWAS studies of severe malaria in children (150).

Another two early studies conducted in Nigeria in 1962 (151) and Liberia in 1963 (152) used infected red blood and infected mosquito bite respectively to induce malaria infection. Both clearly demonstrated acquired immunity showing the innate ability of malaria-exposed individuals to control parasitaemia and limit clinical symptoms (151, 152). These observations were reproduced in modern CHMI studies again using aseptic, purified and cryopreserved sporozoites in Africa. Furthermore, the administration of sporozoites to Kenyan (43, 71), Tanzanian (31) and Gabonese (73, 153) adults at doses that causes 100% infection among HIC volunteers resulted in a proportion of individuals with the ability to limit or completely suppress parasite growth.

Immunological studies confirm the relevance of antibodies to protection as seen in CHMI. The level of antibodies to whole parasites (anti-schizont (71), anti-merozoite (154), anti-ring stage (155)) is independently associated with protection from clinical disease. In addition, the ability of antibodies to induce Fc-mediated antibody effector functions (154–156) further explains protection in CHMI. The breadth of effector functions was shown important as the breadth of Fc function clearly distinguished clinical immune individuals from non-immune individuals (154). Understanding the full profile of antigen specific Fc mediated mechanisms of protection, their rate of acquisition and durability may be important to tailor vaccines with improved potency.

The prioritization of antigens for vaccine development has been challenging as *Plasmodium* parasites have a vast antigenic landscape with considerable diversity. A study conducted in Kenya used a custom protein microarray KILchip expressing >100 immunogenic merozoite surface antigens to screen responses among individuals with demonstrable clinical immunity post challenge (157). This study showed combinations of antigens that may be associated with sterile protection (*manuscript under review*). In parallel, another study explored anti-variant surface antigens (VSA) antibodies (158) and showed that the breadth of VSA was a stronger predictor of protection compared to a single VSA response. Identifying the minimum critical combination of responses needed to achieve sterile immunity by vaccination remains an important scientific goal. The fact that multiple responses are co-acquired over repeated exposures that may not necessary be critical for sterile immunity makes this challenging.

CHMI studies in Africa have also been used to test candidate vaccines, including the whole irradiated sporozoite vaccine evaluated in Tanzania (31) and Mali (72), GMZ2 in Gabon (113) and R21/Matrix-M in Kenya (96). CHMI in Africa in the modern era has relied on cryopreserved sporozoites, which were initially delivered by intramuscular or intradermal injection, but more recently by direct venous injection, which has been shown to be the most efficient method (159). In Kenya, comparisons have been made of R21/Matrix M protection against intradermal injection (ID) vs direct venous inoculation (DVI) of *P. falciparum* sporozoites (PfSPZ Challenge) (96). R21/Matrix-M was highly protective against intradermal inoculation of PfSPZ (i.e. 100%, 12 out of 12) but not protective against PfSPZ challenge by DVI (i.e. 0%, 0 out of 5) (96).

## Ethical considerations learnt for CHMI in Africa

A key ethical consideration that has emerged from CHMI studies in Africa is the value of community engagement. This involves increased communication between communities and research teams during the planning and implementation of studies (160). Combining community involvement with informed consent greatly empowered the decision making of volunteers. A participatory approach acknowledges the cultural context and values of local populations, creating an ethical framework for research (161). Another important ethical consideration is the monetary compensation that is a fair reflection of the volunteers’ commitment to the study. This must be considered carefully to avoid undue inducement among different populations at different economic levels (127). Frameworks to engage with the community to determine adequate compensation have been developed (162, 163).

Improved understanding of CHMI studies among institutional review boards in different African countries has allowed improved regulation. Some African countries have developed specific regulatory guidelines for CHMI studies. This ensures that modern CHMI studies in many African countries are done according to international ethical standards (164). This may be supported by ethics training for researchers and local ethics committees to be able to handle the unique challenges of CHMI studies in Africa (165).

## Conclusions

The use of human infection studies to guide vaccine development and understand pathophysiology of infection is expanding and diversifying to an increasing range of pathogens (11). The malaria parasite includes three main stages of its life cycle in humans which are targets of vaccination, and that are accessible to study through human infection studies, (i.e. controlled human malaria infection). Refinements to CHMI continue to be made with regards to the challenge agent and study conduct. As malaria vaccines are rolled out we predict that there will be increasing reliance on CHMI for the following reasons: a) the currently licensed vaccines will need multiple policy decisions regarding the timing of boosters, new regimens, and manufacturing process updates, and CHMI studies will be needed to confirm efficacy; b) it is likely that multi-stage vaccines will be developed by combining individual components targeting different life cycle stages, and similarly it will not be practical to design field trials that test the efficacy of each component at each stage of clinical development, whereas CHMI trials can be adapted to isolate efficacy for the different life cycle stages as well as optimize vaccine regimen; c) next generation vaccines will need to be compared against existing products (where replacement is proposed) or in addition to standard of care (where addition is proposed), and in either case field trials will become large. CHMI studies will de-risk these approaches; d) ongoing studies of correlates of protection and/or infection, either based on naturally acquired or vaccine induced immunity, will have greater power when the exposure is standardized in CHMI rather than heterogenous in field trials (71, 96). Continued investment in infrastructure, capacity, and the design of CHMI studies tailored for malaria endemic areas will be important for malaria elimination.
